# The Atlanta Medico-Chirurgical Association

**Published:** 1878-04-20

**Authors:** 


### SOCIETY REPORTS.

### THE ATLANTA MEDICO-CHIRURGICAL ASSOCIATION.

REPORTED BY DR. WORD.

Monday Evening, March 18.

Dr. G. G. Crawford in the Chair.

Dr. A. R. Alley asked the views of members relative to the treatment of goiter—having a case under treatment on a girl 16 years of age. The patient was pale and anæmic. The tumor was yet small. The iodine treatment was under trial.

Dr. Roach had found the treatment of goiter very unsatisfactory. Constitutional treatment was indicated in this case, but the prognosis in general he regarded very unfavorable as to the eradication of the disease or entire removal of the tumor.

Dr. W. R. D. Thompson advised the application of a belladonna plaster to the tumor for 48 hours to soften and relax the tissues, to be followed by the persevering use of the iodide of iron internally, to build up the general health.

Dr. J. J. Knott stated that he had treated a case successfully in 1876. There was amenorrhea in the case—uterine derangement is a frequent accompaniment of this disease. He used in the case referred to, the iodide of potash, with bark, and pyrophosphate of iron internally; also used aloes and iron to regulate the catamenia.

Dr. R. C. Word said that in the case reported, being in so young a subject, and the tumor so small there was hope of recovery. He advised the persevering use of iodine ointment to the tumor, and the internal administration of iodoform and iron, as a tonic alterative and resolvent.

Dr. Powell being requested to give his opinion, stated that he did not believe he could throw any additional light upon the subject. He would merely say in reply to the question of Dr. Alley, that the treatment of goiter, especially the local treatment, should depend upon the nature of the enlargement—there being several kinds known as goiter. He believed the best treatment in all cases was the internal administration of iodide of potassium, and the application of the tincture of iodine to the parts, and to be injected in all the forms except the vascular. He was of the opinion, however, that the disease was often hereditary, as he had known it to exist in two and three members of the same family, and had observed that such cases seldom, if ever, recovered. Cases resulting from general bad health were amenable to remedies and were often cured by treatment directed according to the nature and cause of the disease.

Dr. J. J. Knott stated that he thought the disease was not hereditary in any case, and could not recall, in his reading, any authority for the hereditary theory. The disease seemed to be confined, as a rule, to certain localities, particularly in Switzerland and other mountainous countries, and was more likely dependent upon an excess of lime in the water; it might be the absence of salt and iodine, or to other endemic or local influences. He regarded iodine almost as a specific, especially in the early stages of the disease.

Dr. Powell replied that he was aware that authors stated that the disease was very common in the mountainous districts, especially of the Alps, and that it was attributed to some peculiarity of the water, but all agree that this opinion was based upon no satisfactory evidence. He used the word hereditary because it is appropriately applied to all diseases communicated by the parent to the offspring, and that it was an established fact that diseases of the glands could be transmitted in the mysterious process of generation as well as diseases of the lungs, joints, eyes and skin. If his friend Di. Knott will consult the catalogues of the Nosologists, he will find very few diseases that are not capable of such transmission, and properly denominated hereditary.

Dr. E. J. Roach reported a labor case —a face presentation, the mouth presenting. It was a primapara, and the labor continued from Thursday until Monday morning. The waters were discharged on Friday night. He did not see the case until Saturday, a "*granny*" having been in charge. The case righted itself spontaneously on Monday morning. How was this to be accounted for after so long a time? There were circumstances which led him to hope and wait in this case; but are not such results exceedingly rare?

Dr. Powell stated that spontaneous version in face presentation was dependent upon circumstances. The most frequent cause of face presentations, as stated by authors, is obliquity of the womb. They are not face presentations in the beginning, but forehead cases, which are frequently adjusted spontaneously, particularly if the chin presented under the pubis; therefore, the great object in all face presentations should be to bring the chin to the pubic arch, and leave the rest to nature; otherwise, should the vertex present to the pubis and the chin towards the posterior part of the pelvis, efforts should be made to convert it into a vertex position in order to prevent the difficulty that would necessarily occur from throwing a five inch diameter of the head into a four or four and a half inch diameter of the pelvis. But should it not be possible to avoid this position, we must do the best we can; first, attempt to push up the head and bring down the vertex. If labor is far advanced, this maneuvre will seldom succeed, and should the pelvic version be also impossible for the same reason, instruments should be resorted to: first the lever, then the common forceps and crotchet. With these the desired change is often effected, provided the operator is thoroughly acquainted with the mechanism of labor and the movements to be made in each particular case; but in some cases the best directed efforts will prove abortive, and the perforator and embryotomy forceps must be resorted to.

Dr. W. R. D. Thompson stated that such malpresentations were not likely to occur before the entrance of the fetus into the superior strait. He thought that bad handling was the usual cause of such difficulties.

Dr. Knott said he had encountered a similar case to the one mentioned, caused by the manipulations of a midwife. He succeeded in rectifying the position and delivering the child.

Dr. Word reported a case of miscarriage at four and a half months, in which much trouble was encountered in the delivery of the after-birth, and inquired as to the practice of members in such cases. It had been his rule, in all instances where the hemorrhage was excessive, whether the result of a partially detached ovum or placenta, to deliver by placing the heels of the patient to the buttocks, the body being slightly elevated; then by pressure with one hand above the symphisis to force down the womb, and with one or two fingers in the vagina to detach and remove the contents of the uterus. But in the case reported, this plan, by reason of the unusual depth of the pelvis and the firm attachment of the placenta, was found impracticable for the first time in his experience.

Dr. J. J. Knott stated that he thought there was very little risk from retained placenta in abortion. Had known it retained many days without any unfavorable results.

Dr. Thompson concurred with Dr.

Knott. There was no serious danger from retained after-birth in these premature cases; mentioned a case at the fourth month, in which all efforts to deliver the secundines failed, and which was not discharged until the fourteenth day.

Dr. A. R. Alley mentioned a case of abortion at the fourth month—a retained placenta; was attended by a black, grumous, fetid discharge. Portions were detached and removed. Under the use of whisky and ergot, it was all finally expelled. Thinks that there must be much danger from absorption of septic matter in such cases, for which reason, considers the early removal of the placenta important.

Dr. Powell: The placenta is not well developed at so early a stage of labor. In abortion, previous to the fourth month, it is not necessary to make any great effort to deliver the membranes. Certain authors have recommended forceps and scoops for this purpose, but these are now generally discarded. In more advanced cases, or at full time, there might be risk for the placenta to remain longer than a day or two, but in these early cases may be let alone, and antiseptic washes used and chlorate of potash given internally. In the case of Dr. Word, the placenta was probably adherent or partially detached. In such cases, a reasonable effort having failed, I use the tampon. It is safe to use the tampon in such cases, because the womb is small, and cannot, therefore, hold blood enough to endanger the patient. At full time, this would not be the case, and the tampon would not be admissible, and it might be necessary to introduce the hand into the womb, secure its contraction, and deliver the after-birth. In normal cases, unattended by excessive hemorrhage, he did not adopt the practice so fashionable of late of the speedy delivery of the after-birth.

Dr. H. B. Lee differed with Dr. Powell on the point last mentioned: placenta should be delivered at once; concealed hemorrhage sometimes resulted from delay. The parts are less sensitive immediately after the delivery, and less pain is occasioned by the removal. Use pressure upon the womb externally, secure its contraction, follow it down and deliver. If partially detached, all agree that it should be removed. If wholly detached, certainly no harm can result from its removal. It was seldom necessary to introduce the hand into the womb. One or two fingers usually could accomplish the object.

